# Successful Management of Parkinson's Disease Dyskinesia During Local Anesthesia With Dexmedetomidine

**DOI:** 10.7759/cureus.13739

**Published:** 2021-03-06

**Authors:** Takamichi Nakajima, Yasuyuki Suzuki, Noriyuki Miyaue

**Affiliations:** 1 Center for Medical Education and Training, Saiseikai Matsuyama Hospital, Matsuyama, JPN; 2 Anesthesiology, Saiseikai Matsuyama Hospital, Matsuyama, JPN; 3 Neurology, Saiseikai Matsuyama Hospital, Matsuyama, JPN

**Keywords:** parkinson’s disease, dexmedetomidine, dyskinesia, propofol-induced dyskinesia

## Abstract

Dyskinesia is among the most problematic issues for local anaesthesia management in Parkinson’s disease (PD) patients. We present a case of a patient with PD who underwent spinal anaesthesia while utilizing dexmedetomidine (DEX) to reduce dyskinesia during urological surgery. DEX stimulates α₂-adrenergic receptors, which works to reduce the dyskinesia in PD patients.

## Introduction

The main points of concern in the perioperative management of Parkinson's disease (PD) patients are unstable autonomic activity and airway problems due to swallowing disorders. Additionally, levodopa-induced dyskinesia is a common complication among PD patients treated with levodopa [1]. It can be problematic for local anaesthesia management in patients with PD because unexpected, involuntary movements can cause surgical technique complications. Deep sedation and muscle relaxants can easily alleviate dyskinesias; however, use of these medications reduces respiratory function. Although this can be solved by standard airway management if the patient can tolerate positive pressure ventilation, there are concerns regarding the management of some patients who should avoid positive pressure ventilation due to respiratory complications. Positive pressure ventilation is known to cause ventilator-associated lung injury by applying pressure stress to the lungs. This stress can be due to high shear stress at the interface between the atelectasis and the expanded lung, or it can be due to damage caused by high pressure applied directly to vulnerable parts of the lung. We believe that such cases should be managed by local anaesthesia with light sedation under spontaneous respiration.

Regarding the sedative drug choice, dexmedetomidine (DEX) stimulating α₂-adrenergic receptors has been reported to reduce intraoperative involuntary movements during surgery [2]. Here, we describe a case of a patient with PD who received spinal anaesthesia with DEX reducing dyskinesia during surgery, and review the sedative drugs should be chosen in PD patients. Informed consent was obtained from the patient for publication.

## Case presentation

The patient was a 69-year-old woman with a weight and height of 39.0 kg and 143.9 cm, who was scheduled for tension-free vaginal mesh surgery for pelvic organ prolapse. The preoperative examination showed no abnormality in both the electrocardiogram and echocardiogram, and there were no findings of note in the blood tests. Because of the respiratory complications described below, the American Society of Anesthesiologists physical status was Class 3. She developed bradykinesia in her right leg at the age of 56 years. The following year, she was diagnosed with PD and started treatment with levodopa. She gradually exhibited the wearing-off phenomenon and dyskinesia and required frequent oral administration of anti-Parkinson's drugs (a single tablet containing 100 mg of levodopa and 10 mg of carbidopa, consumed six times a day). At the age of 68 years, she was started on levodopa-carbidopa intestinal gel (LCIG) therapy providing continuous levodopa administration via perctaneous endoscopic gastrojejunostomy tube, which alleviated her motor fluctuations such as the wearing-off phenomenon. She needed assistance with some activities of daily living and classified as having Stage 4 PD on the Hoehn and Yahr scale. Treatment level of LCIG was a morning dose of 7.0 mL/h, a continuous dose of 2.0 mL/h, and extra doses of 1.0 mL given once a day. She continued LCIG therapy during the perioperative period; however, she often showed choreiform dyskinesia of the trunk and limbs. The patient was deemed safe for surgery by the neurologist.

Furthermore, she had previously suffered from a pneumothorax caused by lymphangioleiomyomatosis. Anaesthesiologists often avoid general anaesthesia because of the risk of intraoperative respiratory complications, such as pneumothorax due to pressure injury [3], in lymphangioleiomyomatosis patients and manage them with local anaesthesia. However, local anaesthesia methods, especially spinal anaesthesia, do not suppress the upper limb dyskinesia, which can interfere with surgical progress. Unexpected body movement of the patient is a threat that can cause damage by surgical instruments. Therefore, we decided to carry out spinal anaesthesia combined with DEX to suppress dyskinesia.

Spinal anaesthesia was performed by injecting 2.5 mL of 0.5% hyperbaric bupivacaine into the L3-L4 interspace. After confirming the sensory blockade up to T10, we began intravenous injecting DEX at 0.7 µg/kg/hour. The patient gradually fell asleep and there were no intraoperative respiratory, circulatory, and neurological complications, including involuntary movements. We stopped injecting DEX after the surgery and her dyskinesia returned gradually within the hour.

## Discussion

For local anaesthesia management of patients with PD presenting dyskinesia, involuntary movements must be suppressed to ensure safe surgery. This dyskinesia is mostly caused by levodopa, which is termed levodopa-induced dyskinesia (LID). A previous study reported that 27.9% of patients with de novo PD had experienced LID for a median duration of 4.6 years [4]. Generally, involuntary movements, including dyskinesia, disappear when the patients fall asleep. Previous studies have reported that supersensitive γ-aminobutyric acid type A (GABAA) receptors in the globus pallidus pars interna are implicated in the neurochemical mechanisms of dyskinesia in patients with PD who take levodopa [5]. It was recently reported that administration of a GABA antagonist suppressed LID in a rat model of Parkinson's disease [6]. We assumed that unconsciousness induced by GABAA receptors may cause dyskinesia, depending on the anaesthesia depth. Therefore, appropriate sedation needs to be chosen in patients with PD who present dyskinesia.

Here, we focus on some of the most common anaesthetic agents. Propofol and midazolam are popular anaesthetic agents that stimulate GABAA receptors to induce unconsciousness. Moreover, propofol suppresses N-Methyl-D-aspartate receptors. In contrast, DEX works by stimulating α₂-adrenergic receptors. There have been several reports of propofol-induced dyskinesia (PID); however, its mechanism remains unclear. One study suggested that propofol may potentiate aminobutyric acid-mediated transmission in the thalamocortical outflow [2]. 

Midazolam was more useful than propofol for alleviating LID during local anaesthesia and it stimulates the GABA receptors to regulate the thalamus and brainstem through the basal ganglia [7]. However, midazolam has a longer half-life and slightly poorer adjustability than propofol, so care should be taken with dosage.

Contrastingly, it has been reported that midazolam usage for local anaesthesia in patients with PD induced dyskinesia or involuntary movement [2]. Although there have been no other reports of midazolam-induced dyskinesia in patients with PD, several studies have reported midazolam-induced abnormal involuntary movements in patients with non-parkinsonian diagnoses [8,9]. Therefore, we are unsure if midazolam is appropriate for local anaesthesia in patients with PD.

DEX may supersede propofol and midazolam since it induces sedation by acting on α₂-adrenergic receptors. DEX has been reported to control PID during brain surgery in patients with PD [2]. Another study reported that α₂-adrenergic receptor agonists and antagonists reduce and potentiate the levodopa effect, respectively [10], which might explain dyskinesia alleviation by DEX in patients with PD.

Generally, respiratory suppression induced by DEX is lesser than that induced by other sedation agents [11]. There are also two reports that the combined use of DEX with local anaesthesia prolonged the effect of the local anaesthetic [12,13]. Although it is not a common method of administration and is still in the research stage, there is a report that DEX administered into the subarachnoid region of the spinal cord produced a mild sedative effect while enhancing the effects of spinal anaesthesia [14]. Therefore, we suggest that DEX is among the most suitable sedatives for local anaesthesia in patients with PD who present dyskinesia.

## Conclusions

In conclusion, we present a case of local anaesthesia management using DEX in a patient with PD who presented dyskinesia. DEX has a sedative effect by stimulating α₂ receptors, not by stimulating GABAA receptors that may induce LID. Stimulation of α₂ receptors also has the advantage of attenuating the effects of levodopa. It has the potential to be the best sedative for perioperative PD patients because it is extremely resistant to respiratory depression and inhibits LID. We believe that this case report of perioperative management of dyskinesia without respiratory depression supports this possibility.

## References

[1] Damiano AM, McGrath MM, Willian MK (2000). Evaluation of a measurement strategy for Parkinson's disease: assessing patient health-related quality of life. Qual Life Res.

[2] Deogaonkar A, Deogaonkar M, Lee JYK, Ebrahim Z, Schubert A (2006). Propofol-induced dyskinesias controlled with dexmedetomidine during deep brain stimulation surgery. Anesthesiology.

[3] Peterfreund RA, Luman E, Martuza RL (2012). Anesthesia for suboccipital craniotomy in a patient with lymphangioleiomyomatosis: a case report. Case Rep Pulmonol.

[4] Eusebi P, Romoli M, Paoletti FP, Tambasco N, Calabresi P, Parnetti L (2018). Risk factors of levodopa-induced dyskinesia in Parkinson’s disease: results from the PPMI cohort. NPJ Parkinsons Dis.

[5] Calon F, Di Paolo T (2002). Levodopa response motor complications—GABA receptors and preproenkephalin expression in human brain. Parkinsonism Relat Disord.

[6] Nishijima H, Mori F, Arai A (2020). GABA storage and release in the medial globus pallidus in L-DOPA-induced dyskinesia priming. Neurobiol Dis.

[7] Shibuya M, Hojo T, Hase Y, Fujisawa T (2018). Conscious sedation with midazolam intravenously for a patient with Parkinson’s disease and unpredictable chorea-like dyskinesia. Br J Oral Maxillofac Surg.

[8] Stolarek IH, Ford MJ (1990). Acute dystonia induced by midazolam and abolished by flumazenil. BMJ.

[9] Davies A (2000). Midazolam-induced dyskinesia. Palliat Med.

[10] Haapalinna A, Leino T, Heinonen E (2003). The alpha 2-adrenoceptor antagonist atipamezole potentiates anti-Parkinsonian effects and can reduce the adverse cardiovascular effects of dopaminergic drugs in rats. Naunyn Schmiedebergs Arch Pharmacol.

[11] Koroglu A, Teksan H, Sagr O, Yucel A, Toprak H, Ersoy O (2006). A comparison of the sedative, hemodynamic, and respiratory effects of dexmedetomidine and propofol in children undergoing magnetic resonance imaging. Anesth Analg.

[12] Furqan A, Mohsin MU, Sattar MK, Khan AA, Shahid M, Fayyaz A (2019). Intravenous dexmedetomidine has synergistic effect on subarachnoid block with hyperbaric bupivacaine. Cureus.

[13] Samar P, Dhawale TA, Pandya S (2020). Comparative study of intravenous dexmedetomidine sedation with perineural dexmedetomidine on supraclavicular approach brachial plexus block in upper limb orthopaedic surgery. Cureus.

[14] Sharma A, Varghese N, Venkateswaran R (2020). Effect of intrathecal dexmedetomidine versus intravenous dexmedetomidine on subarachnoid anesthesia with hyperbaric bupivacaine. J Anaesthesiol Clin Pharmacol.

